# Combined Diet and Supplementation Therapy Resolves Alopecia Areata in a Paediatric Patient: A Case Study

**DOI:** 10.7759/cureus.11371

**Published:** 2020-11-07

**Authors:** Cliff J Harvey

**Affiliations:** 1 Clinical Nutrition, The Holistic Performance Institute, Auckland, NZL

**Keywords:** alopecia areata, nutrition, vitamin d, zinc, vitamin a, supplementation

## Abstract

Alopecia areata (AA) is a common autoimmune condition resulting in spot baldness and, rarely, more extensive hair loss. There is an association between both the incidence and the severity of AA and several micronutrients, including vitamin D and zinc. This case reports an eight-year-old male diagnosed with AA and treated with a diet and supplemental regimen based on unrefined foods, rich in vitamins A and D, zinc, and supplemented with a multi-nutrient, zinc sulfate, and fish oil with vitamin D. Complete remission of AA was achieved within five months.

## Introduction

Alopecia areata (AA), sometimes known as ‘spot baldness’ is a condition in which hair is lost, typically in patches over some of the body but occasionally becoming more severe and affecting the entire body. The condition affects around 2% of people at some time in their life [1]. It is believed to be an autoimmune disease with some inheritability, and different immune‐cell lines, including plasmacytoid dendritic cells, natural killer cells, and T cells, along with key molecules, such as interferon‐γ, interleukin‐15, MICA and NKG2D, and CD8 T cells have been identified as contributing to the autoimmune process [2-3]. Genome-wide association studies provide evidence for the involvement of both innate and acquired immunity in the pathogenesis of AA [3]. There is also evidence that AA is associated with oxidative stress [4].

## Case presentation

An eight-year-old male presented for nutritional and supplemental advice secondary to a diagnosis of alopecia areata. Initial blood tests were within normal ranges for C-reactive protein, fasting glucose, ferritin, liver enzymes, electrolytes, and all complete blood count measures. Coeliac disease was ruled out due to tissue transglutan immunoglobulin A (IgA) < 0.5 U/ml and total IgA 0.4 g/L. Thyroid disease was not suspected, as thyroid peroxidase antibodies were within normal ranges (37 U/ml) and thyroid-stimulating hormone was 2.6 mIU/L.

Further blood tests were requested for vitamin B12 and folate, magnesium, serum zinc, rheumatoid factors, anti-nuclear antibodies, along with a repeat complete blood count. Except for marginally low neutrophils and monocytes, no abnormalities were noted. Serum zinc returned a ‘normal’ range result of 0.68 mcg/ml but this was below the suggested lower threshold of >0.7 mcg/ml for functional outcomes in boys of this age (6-9 yrs.) [5]. Similarly, while the 25 hydroxyvitamin D (25(OH)D) result of 90 nmol/L was within reference ranges, it has been suggested that the lower threshold for optimum 25(OH)D status is >100 nmol/L [6].

Clinical course

The patient’s parents were provided with advice to increase zinc and vitamin A and D-rich foods, avoid gluten and dairy where able, and to focus on a diet that prioritizes foods in their natural forms in preference to highly processed ‘packaged’ foods. Also, a supplementation regimen consisting of a multi-nutrient (multi) powder rich in vitamins A and D3, zinc, and secondary antioxidant nutrients (Kids Good Stuff, Nuzest, Potts Point NSW, Australia), a zinc sulfate supplement (zinc drops, Clinicians Ltd., Australia), and fish oil with added vitamin D (fish oil + vitamin D, Melrose, Victoria, Australia) was provided (key nutrient summary in Table 1), along with lifestyle advice to get outside for 5-10 min per day without sunblock on. A full list of the multi-nutrient ingredients can be found in Appendix 1.

**Table 1:** Amounts of supplemental nutrients provided RE: retinol equivalent; DHA: docosahexaenoic, EPA: eicosapentaenoic acid

	Amount provided in the supplements	Total supplemental dose per day
Vitamin A (μg RE)	Multi: 400	400
Vitamin D3 (μg)	Multi: 10, Fish oil: 12.5	22.5 (900 iu)
Zinc (mg)	Multi: 6, Zinc drops: 4	10
Omega 3 fatty acids (mg)	Fish oil: DHA – 273, EPA – 410	683 (total n-3 fatty acids)

A sample of the dietary advice is provided below.

Vitamin A: Fish oil, liver, oily fish (herring, sardines, salmon, cod), eggs, chicken Also: kumara, pumpkin, carrots, mangos, apricots, broccoli, full-fat yogurt and milk*

Vitamin D: Cod liver oil, liver, fatty fish, full-fat milk and yogurt*

Note: It is imperative to use full-fat foods, as vitamins A and D are fat-soluble.

Zinc: Oysters, beef, pork, chicken, pumpkin seeds, full-fat yogurt*, almonds, fish

Note: Excessive intake of cereal grains might inhibit some zinc absorption.

*We are reducing/avoiding dairy in this phase, but these foods can be reintroduced to test tolerance when symptoms abate.

**Figure 1:**
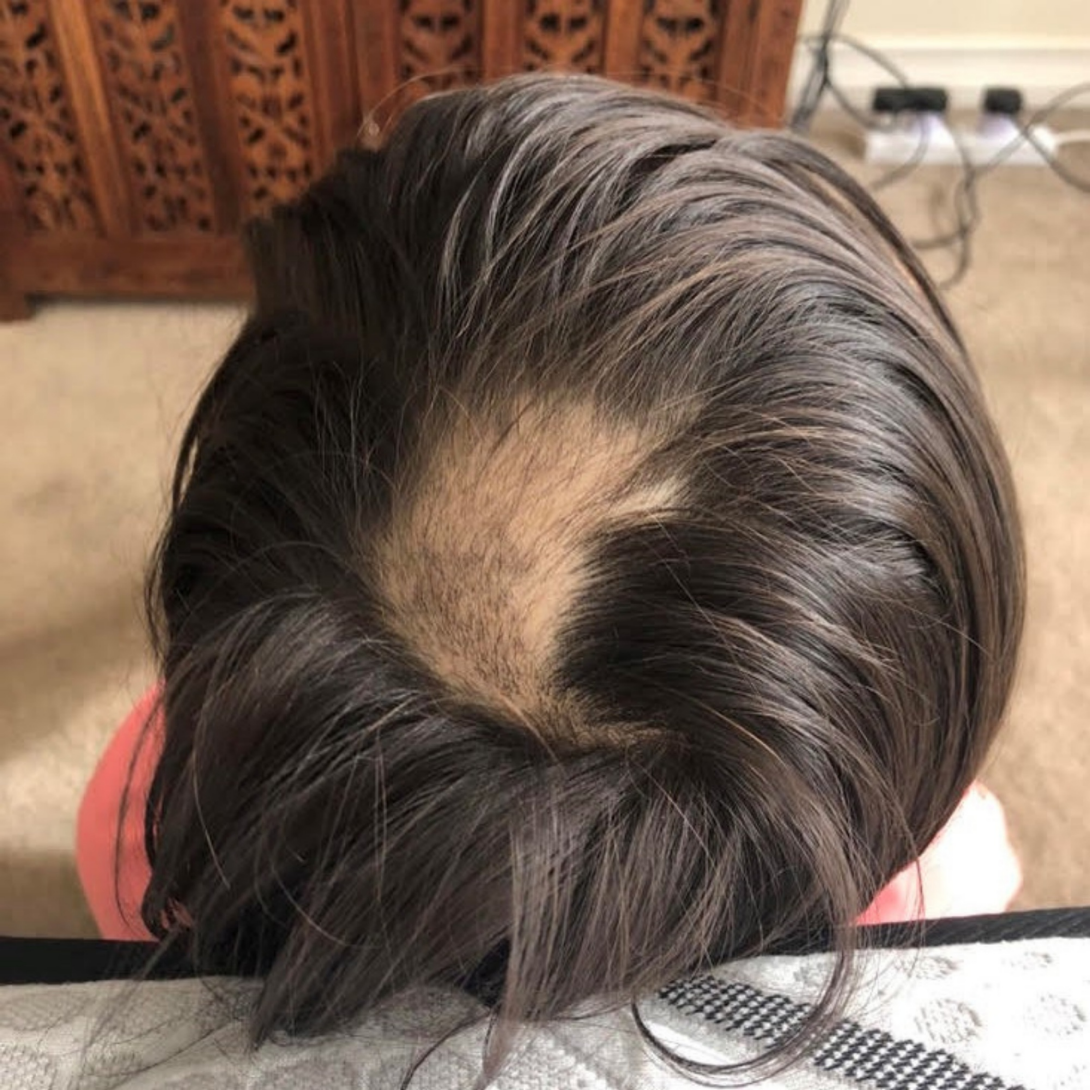
Spot baldness one week after the initial consultation

On presentation, the client had experienced severe hair loss with the involvement of the eyelashes, eyebrows, and mostly spot baldness on the crown of the head (Figure 1).

**Figure 2:**
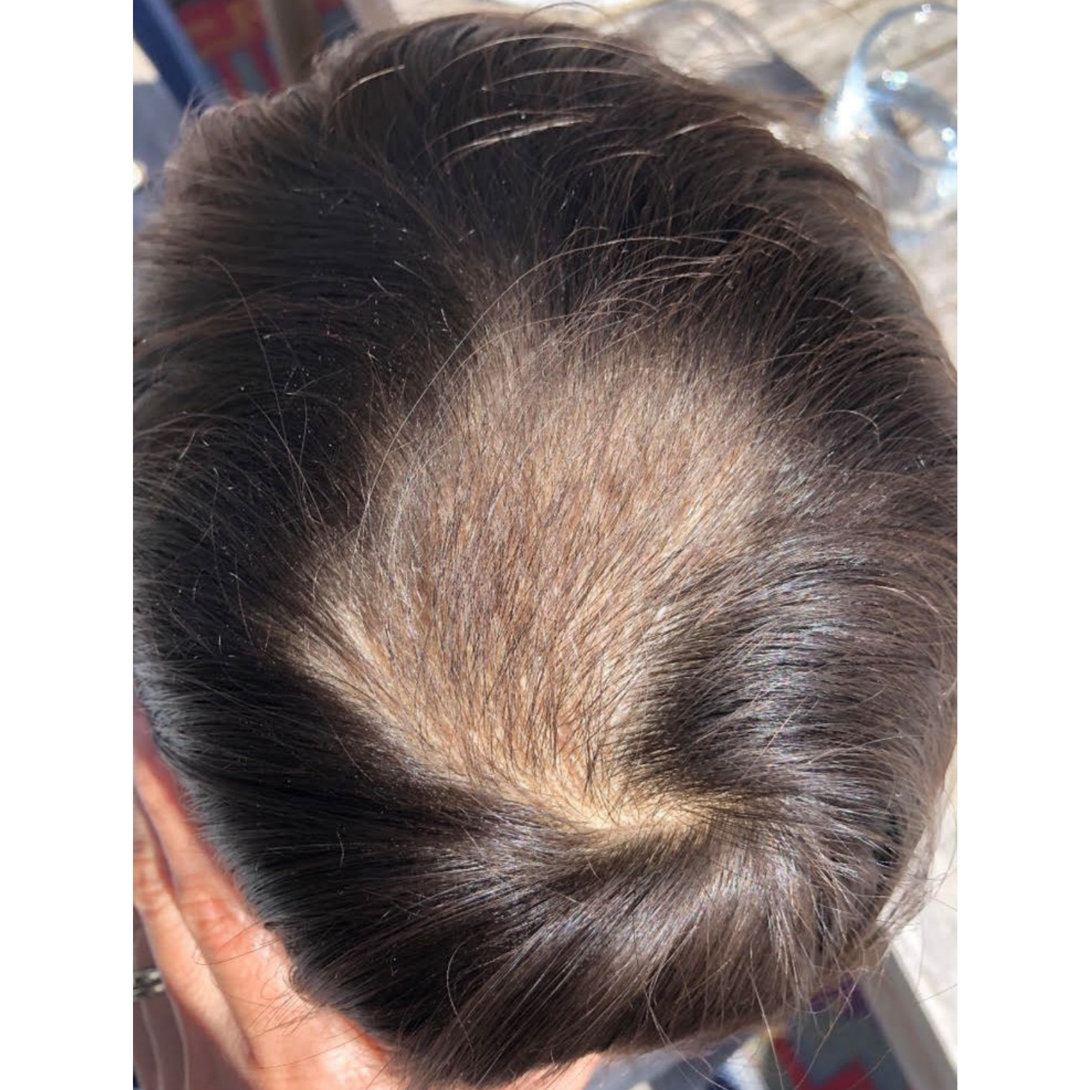
Patient hair regrowth at two months

After following the prescribed dietary regimen for two months, the patient's hair can be seen to be regrowing in Figure 2.

**Figure 3:**
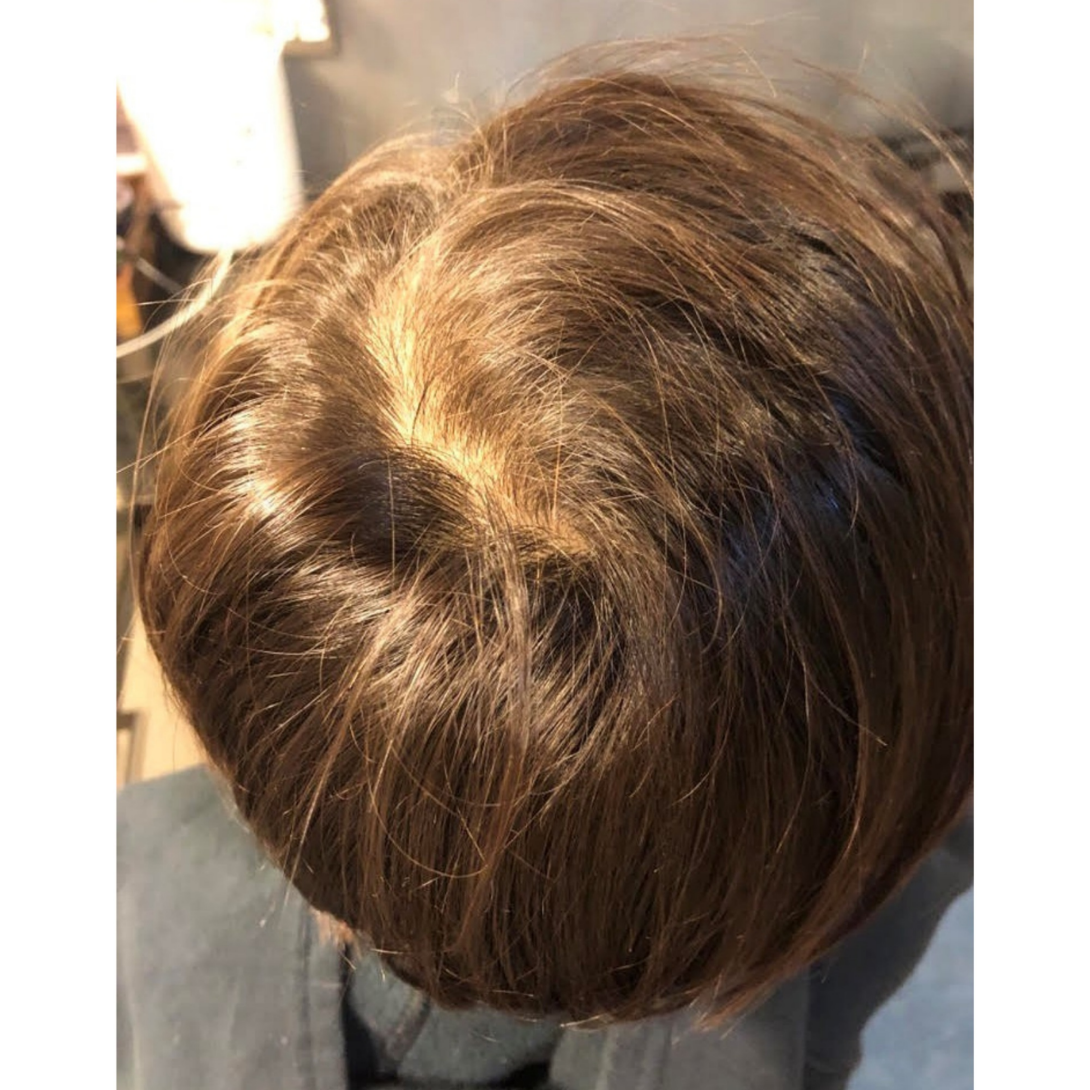
Patient hair regrowth five months following commencement of the diet and supplement regimen

Five months following commencement of the diet and supplement regimen, the hair on the crown was completely recovered and the patient's mother reported that the eyelashes and eyebrows were growing back (Figure 3). Approximately two weeks after commencing the diet and supplementation regimen, the patient exhibited a limited, minor popular rash on the cheeks, which resolved shortly afterwards and by August, there were no signs of any eczema-like skin conditions.

## Discussion

Patients with AA tend to have lower serum vitamin D, zinc, and folate levels as compared to controls, and evidence also suggests that vitamin A status may help modify the disease [7].

In particular, vitamin D status appears to be markedly different in patients with AA. Vitamin D levels (serum 25(OH)D) in patients with alopecia have been demonstrated to be significantly lower than in healthy controls (11.84 ± 6.18 vs 23.57 ± 9.03 ng/mL p < 0.001) [8], and the prevalence of vitamin D deficiency significantly higher [9]. Disease severity in AA also appears to be inversely correlated to vitamin D status [8,10-11]. While there have not been significant genetic differences (gene polymorphisms) shown for the vitamin D receptor (expressed in hair follicles) between alopecia and controls [12], tissue vitamin D receptor levels in tissue are lower in alopecia versus controls [13] and this is associated with increased inflammation but not vitamin D levels or severity and pattern of illness [14].

Serum zinc levels have also been demonstrated to be lower in people with AA as compared to health controls (t = 4.206, p = 0.001) and are associated with the severity of the condition (r = -0.573, p = 0.001) [15]. Other research has also demonstrated that alopecia patients have significantly lower zinc levels than controls [16-17]. For example, Orecchia and colleagues observed a mean plasma zinc level of 74.2 versus 95.5 μg/100 ml in cases as compared to controls respectively [17]. And while some studies have shown no significant difference in serum zinc levels between alopecia patients and controls, subgroup analysis revealed lower zinc levels in those with greater disease severity [18].

While this case has focussed on key nutrients with demonstrable associations with either disease incidence or severity, others such as biotin [19] have also been implicated and warrant further research.

Therefore, it is likely that nutrient-repletion is a critical and yet often overlooked strategy in the treatment of AA.

## Conclusions

Vitamin D and zinc (and vitamin A) are critical to immune function and may provide an adjunct treatment option for AA. Insufficiency of these key micronutrients, whether primary or secondary to genetic polymorphisms, is linked to both the incidence and severity of AA. Normal cycles of relapse and remission common to autoimmune conditions, the placebo effect, or lifestyle factors may have contributed to or been responsible for the remission seen in this case and ongoing observation and follow-up will help to ascertain this. However, it demonstrates that a diet, excluding common autoimmune trigger foods and replete in key micronutrients, holds promise for the treatment of this condition, and research is warranted to investigate this hypothesis further.

## Appendices

**Table 2:** Nutritional information of Nuzest Kids Good Stuff RE: retinol equivalent; TE: tocopherol equivalent Percent of average recommended daily intake for children ages 4-14

	Amount provided in the multi-nutrient	% RDI
Vitamin A	400 μg RE	63
Thiamin (B1)	2.2 mg	244
Riboflavin (B2)	3.4 mg	354
Vitamin B3	10 mg	81
Pantothenic acid	5 mg	109
Vitamin B6	1.6 mg	160
Folate	200 μg	67
Vitamin B12	5 μg	278
Vitamin C	100 mg	261
Vitamin D3	10 μg	200
Vitamin E	10 mg TE	122
Vitamin K	80 μg	178
Biotin	60 μg	280
Calcium	206 mg	21
Magnesium	50 mg	18
Zinc	6 mg	83
Iron	1.4 mg	13
Manganese	1 mg	34
Copper	0.3 mg	25
Selenium	21 μg	40
Chromium	20 μg	83
Iodine	75 μg	63
